# Case report: challenges in monitoring and treatment of anthracycline induced cardiotoxicity in young adults with osteosarcoma

**DOI:** 10.1186/s40959-022-00145-y

**Published:** 2022-11-15

**Authors:** Julius C. Heemelaar, Jeroen Janson, Jerry Braun, Frank M. Speetjens, Michiel A. J. van de Sande, Juan D. V. Hugo, Daniela Q. C. M. Barge-Schaapveld, Saskia L. M. A. Beeres, Laurens F. Tops, Hans Gelderblom, M. Louisa Antoni

**Affiliations:** 1 Department of Cardiology, Heart Lung Centre, Leiden University Medical Center, Albinusdreef 2, 2333 ZA Leiden, Zuid-Holland the Netherlands; 2 Department of Intensive Care Medicine, Leiden University Medical Center, Leiden, Zuid-Holland The Netherlands; 3 Department of Thoracic Surgery, Leiden University Medical Center, Leiden, Zuid-Holland The Netherlands; 4 Department of Medical Oncology, Leiden University Medical Center, Leiden, Zuid-Holland The Netherlands; 5 Department of Orthopedic Surgery, Leiden University Medical Center, Leiden, Zuid-Holland The Netherlands; 6 Department of Clinical Genetics, Leiden University Medical Center, Leiden, Zuid-Holland The Netherlands

**Keywords:** Osteosarcoma, Doxorubicin, Cardiotoxicity, Heart failure, Case report, Left ventricular assist device

## Abstract

Neo(adjuvant) systemic treatment regimens containing anthracyclines such as doxorubicin cause a significant risk of heart failure. These regimens are one of the corner stones of osteosarcoma treatment, and therefore several guidelines are in place to steer cardiotoxicity monitoring through baseline risk stratification and cardiac surveillance during and after completion of cancer therapy.

Importantly, baseline risk stratification modules are dependent on age, prior cardiovascular disease and cardiovascular risk factors. Because the majority of osteosarcoma patients are below 30 years of age these criteria rarely apply and most patients are assigned to low or medium risk categories, whereas cardiovascular complications have profound impact on morbidity and mortality in this young population. Therefore, cardiac surveillance is very important in this group for timely detection of cardiotoxicity. Moreover, when severe cardiotoxicity that requires advanced heart failure treatment occurs, a cancer diagnosis has significant implications on treatment options, i.e. mechanical circulatory support and heart transplantation.

These challenges are presented in this case of a patient without clinical risk factors admitted with cardiogenic shock requiring advanced heart failure treatment within 1 month after completion of doxorubicin containing chemotherapy for the treatment of high grade osteosarcoma.

**Supplementary Information:**

The online version contains supplementary material available at 10.1186/s40959-022-00145-y.

## Introduction

The main stay of osteosarcoma treatment – the most common primary malignant bone tumor among children, adolescents and young adults (AYAs) – is surgery and high intensity (neo)adjuvant systemic treatment that contain anthracyclines [1]. It has been known for decades that anthracyclines cause a substantial risk of cardiotoxicity resulting in heart failure [2, 3]. Based on currently available literature the cumulative incidence of heart failure after osteosarcoma treatment is estimated 2–14% depending on the definition of the endpoint [4–8].

However, predicting which osteosarcoma patients will develop cardiotoxicity during and after treatment is challenging. There are several guidelines and position statements for baseline risk assessment and surveillance, but most risk stratification models primarily focus on traditional cardiovascular risk factors (i.e. hypertension, hypercholesteremia, diabetes mellitus, and smoking), prior cardiovascular disease and cumulative anthracycline dose [9–11]. Patients with osteosarcoma are mostly of young age and infrequently have any comorbidities or risk factors. A risk factor that nearly all osteosarcoma patients share is high cumulative dose of anthracyclines as target dose for osteosarcoma is > 400 mg/m^2^ [12]. Thus, cardiac surveillance with imaging and cardiac biomarkers during and after treatment may play an important role in detection of cardiotoxicity in this population. Unfortunately, there is no consensus on the role and timing of routine imaging and cardiac biomarkers. Therefore, cardiotoxicity monitoring of osteosarcoma patients is inefficacious and inefficient.

Secondly, when severe cardiotoxic complications occur, cancer significantly limits the treatment options for advanced heart failure as a recent cancer diagnosis is a relative contra-indication for heart transplant [13].

We aim to exemplify these challenges through presenting a unique case of a patient without patient-related high risk factors for cardiotoxicity admitted with cardiogenic shock requiring advanced heart failure treatment within 1 month after completion of chemotherapy for the treatment of high grade osteosarcoma.

## Timeline

**Figure:**
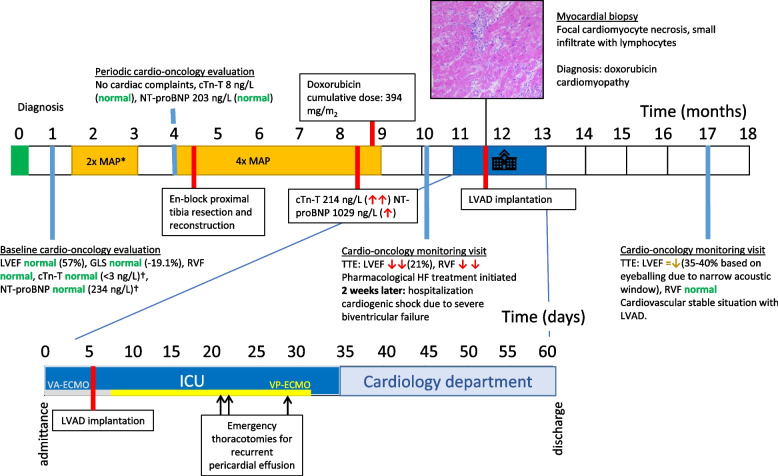
*one cycle of MTX omitted. †normal values: cTn-T < 14 ng/L, NT-proBNP < 247 ng/L. cTn-T = cardiac troponin T; ICU = intensive care unit; HF = heart failure; LVAD = left ventricular assist device; LVEF = left ventricular ejection fraction;MAP = methotrexate/doxorubicin (Adriamycin)/cisplatin; MTX = methotroxate; NT-proBNP = N-terminal pro-brain natriuretic peptide; RVF = right ventricular function; TTE = transthoracic echocardiogram; VA-ECMO = veno-arterial extracorporeal membrane oxygenation; VP-ECMO = pulmonary artery ECMO cannulation.

## Case

A 32-year-old female with recent diagnosis of high grade osteosarcoma was admitted to the emergency room of our institution with cardiogenic shock due to severe biventricular failure within 1 month after finalizing the last chemotherapy cycle.

### Patient information

One year prior to hospitalization she was diagnosed with a telangiectatic osteosarcoma of the proximal left tibia. Past medical history was unremarkable. Family history included one 2^nd^ degree relative who died at 47 years of age following colon cancer, meeting criteria for Li-Fraumeni like syndrome. However no *TP53* mutation was detected. Her treatment plan consisted of 2 cycles of MAP (2 × methotrexate/doxorubicin/cisplatin) neoadjuvant systemic therapy, en-block proximal tibia resection with clear margins and endoprosthesis reconstruction of the knee joint, and 4 cycles of MAP adjuvant systemic treatment according to the standard EURAMOS schedule [12]; See Timeline. The cumulative dose of administered doxorubicin was 394 mg/m^2^. The resection material did not contain vital tumor cells, which is a marker for favorable oncological prognosis. The oncology treatment team estimated the 5-year overall survival at 80% and progression free survival at 60% in this patient.

### Clinical findings

The baseline cardiac evaluation at the cardio-oncology clinic of our institution showed no high-risk features for cardiotoxicity – aside of high dose of anthracyclines—according to the most recent guideline at time of presentation [11] and most recent guideline [10] See Table 1. She had no history or symptoms of cardiovascular disease and no traditional cardiovascular risk factors. Moreover, there was no family history for cardiomyopathy or sudden cardiac death. A 12-lead electrocardiogram showed normal sinus rhythm at 69 beats/minute without Q waves, with a normal QRS width and no repolarization abnormalities. The key parameter of left ventricular function – left ventricular ejection fraction (LVEF)—was normal at 57%. Furthermore, a marker for subclinical left ventricular dysfunction—global longitudinal strain (GLS) – was normal with -19.1%. No further abnormalities were observed on echocardiographic examination and in the levels of biochemical markers cardiac troponin T (cTn-T) and N-terminal pro-brain natriuretic peptide (NT-proBNP).

**Table 1:** High risk features for anthracycline induced cardiotoxicity in case study and recommended cardiotoxicity monitoring strategy

ASCO (2017) *Followed at time of case*	Classify as high risk if.	
	Patient-related factors^a^	Treatment-related factor
	Older age at treatment (≥ 60 years) (**X**) Multiple cardiovascular risk factors (e.g. smoking, hypertension, diabetes, obesity) (**X**) Underlying cardiovascular disease (**X**) Reduced or low-normal LVEF (50–54%) before anticancer treatment (**X**)	High-dose anthracycline ≥ 250 mg/m^2^ (**Y**) High dose thoracic irradiation ≥ 30 Gy (**X**)
	Cardiotoxicity risk: high Follow up recommendation: echocardiogram before start of treatment. In case of clinical signs of cardiac dysfunction echocardiogram in conjunction with serum cardiac biomarkers. Routine surveillance imaging may be offered	
ESMO (2020) *Published after case*	Patient related factors	Baseline risk exam
	Age < 10 or > 75 years (**X**) Previous anthracycline-based treatment and/or chest radiotherapy (**X**) Previous combined treatment trastuzumab and anthracyclines (**X**) Prevalent hypertension, smoking, diabetes (**X**)	Elevated cardiac biomarkers before initiation of anticancer therapy (**X**) Baseline LVEF < 50% (**X**)
	Cardiotoxicity risk: low Follow up-recommendations: periodic measurement of cardiac biomarkers (every 3–6 weeks or after each cycle), echocardiogram beyond doxorubicin cumulative dose 250 mg/m^2^ and at end of therapy	

^a^in combination with anthracycline or trastuzumab treatment

During treatment the patient had no symptoms or signs of heart failure. However, routine measurement showed an elevated cTn-T (214 ng/L, normal range: < 14 ng/L) and NT-proBNP (1029 ng/L, normal range: < 249 ng/L) before the last cycle of doxorubicin. Because the patient was considered a low-risk patient, had a good functional status and there were no clinical signs of heart failure, treatment was continued as this is of large importance for the prognosis of high-grade osteosarcoma. Cardiac remodeling therapy was not initiated due to low blood pressure and the cardiotoxicity monitoring visit was performed after the final chemotherapy cycle, as previously scheduled. At this visit she still had no symptoms of heart failure, but echocardiographic examination revealed a severely reduced LVEF of 21% with an intracavitary thrombus and concurrent poor right ventricular function; See Supplementary Video. Pharmacological heart failure treatment was initiated, however only low dosage of losartan 25 mg/day was tolerated due to symptomatic hypotension. Anticoagulation was started with phenprocoumon for the treatment of the intracavitary thrombus. Unfortunately, within days she developed progressive dyspnea and hypotension. She was presented at the emergency department less than two weeks later.

### Diagnostic assessment: Intensive care unit admittance due to cardiogenic shock

On the day of hospital admission, she had central cyanosis, severe fatigue, dyspnea and anuria. Upon physical examination her blood pressure was 92/50 mmHg with a heart rate 136/min, temperature of 37.0ºC with signs of pulmonary congestion, which was confirmed by chest x-ray. Laboratory tests showed lactic acidosis (pH = 7.29, lactate = 7.2 mmol/L), liver function disorders (alanine aminotransferase = 790 IU/L, aspartate aminotransferase = 1009 IU/L), and prolonged activated partial thromboplastin (67.1 s). The kidney function was preserved (estimated glomerular filtration ratio > 60 mL/min/1.73m^2^). There was imminent organ failure due to cardiogenic shock and congestion. Inotropic support using dobutamine was unable to prevent further deterioration of the clinical condition. Therefore, mechanical circulatory support through veno-arterial extracorporeal membrane oxygenation (VA-ECMO using peripheral canulation in femoral artery) was initiated and the patient was admitted to the intensive care unit (ICU).

### Therapeutic intervention: advanced heart failure management

In patients with acute heart failure refractory to pharmacological treatment mechanical circulatory support is indicated to achieve hemodynamic stabilization. If cardiac function does not recover, heart transplant should be considered [14]. However, in patients with a cancer diagnosis, a cancer-progression free period of 5 years is generally required to apply for heart transplant listing in the Netherlands. Therefore, a left ventricular assist device (LVAD) was implanted with temporary pulmonary artery ECMO cannulation for right ventricular support (VP-ECMO). Listing for heart transplant will be evaluated in the following 5 year period.

The LVAD implantation was performed 8 days after hospital admittance under antimicrobial treatment with meropenem and vancomycin because of elevated inflammatory parameters, which was discontinued after persistent negative blood cultures. Post-operatively 3 repeat thoracotomies were performed in the course of 2 weeks for recurrent cardiac tamponade. The hemodynamic status improved after these episodes and the VP-ECMO could be slowly weaned and explanted 1 month after ICU admission. The patient was transferred to the Coronary Care Unit where further recovery was without any complications. She was discharged from our institution to a rehabilitation center after a 62 day stay.

### Follow-up and outcomes

Three months after discharge cardiac function improved with LVAD support. DNA analysis of 62 genes associated with cardiomyopathy did not reveal any underlying pathogenic mutations: including Lamin A/C and TTN gene mutations [15]. At present, the patient is still undergoing regular follow-up at the medical oncology department and cardiology without notable adverse events 10 months after LVAD implantation and 22 months after osteosarcoma diagnosis.

## Discussion

This case of severe anthracycline induced heart failure reveals important learning points. First, while there was a low suspicion of cardiotoxicity due to absence of heart failure symptoms during treatment and no risk factors aside of high doxorubicin dose at baseline risk assessment, the patient presented with fulminant heart failure and cardiogenic shock. This highlights both importance and difficulty of cardiotoxicity monitoring in AYAs. Second, when severe cardiotoxicity occurs in AYAs, heart transplantation – the preferred advanced heart failure treatment option in this age category – is generally contraindicated up to five year cancer progression free survival. Considering a substantial complication rate (e.g. thrombosis, bleeding, infection) of the alternative – LVAD implantation—this has a significant impact on morbidity and quality of life specific in relation to AYAs with a recent malignancy.

Currently, no consensus exists on both the cardiotoxicity monitoring during anthracycline treatment and the duration of monitoring after treatment. For example, this patient was classified as high risk at baseline according to ASCO guideline, because of high anthracycline dose. [16, 17], In the most recent guideline published after the case – the 2020 ESMO guideline – cumulative dosage is not mentioned as high risk factor. See Table 1. Furthermore, monitoring symptoms relating to heart failure and periodic assessment of biomarkers for cardiac injury play a role in highlighting patients who need prompt reassessment of cardiac function. Because the patient had no cardiovascular complaints during chemotherapy, had both normal cardiac troponin and normal NT-proBNP at baseline and after 250 mg/m^2^ of doxorubicin (3–8 ng/L and 234–203 ng/L, respectively; see Timeline), reassessment of cardiac function was only performed at the end of treatment, as initially planned. At that moment a severely reduced biventricular function was diagnosed although there still were no symptoms or signs of heart failure. Opposed to the 2017 ASCO guideline, the 2020 ESMO guideline recommends an echocardiogram after a cumulative doxorubicin dose of 250 mg/m^2^. While this may have revealed a decreased left ventricular function, this is unlikely because cardiac biomarkers at this time were still normal. Finally, it is important to note that generally accepted cut-offs for cardiovascular biomarkers have not been validated and there is currently non consensus for cut-off values for patients undergoing cancer treatment.

Clinical trials on initiating cardioprotective medication with ACE-converting enzyme inhibitors or betablockers after detection of increased cardiac biomarkers show inconclusive results. Cardinale et al. showed in 2006 that patients randomized to enalapril in response to an increased cardiac troponin I had lower rate of echocardiographic cardiotoxicity compared to patients who did not receive enalapril (0% vs 43% respectively) [18]. Conversely, The ICOS-ONE trial did not show a benefit in troponin-triggered initiation of cardioprotective therapy compared to routinely initiating cardioprotective therapy at baseline in preventing cardiotoxicity defined as LV dysfunction assessed by echocardiography [19]. In this case the patient had low blood pressure and therefore only low dose heart failure medication was tolerated. The clinical impact of this low dose medication on recovery of LV function is uncertain. Opposed to cardiac troponin, the association between elevated levels of NT-proBNP in LV-dysfunction in patients undergoing cancer treatment is less certain. In a meta-analysis of 10 individual by *Michel *et al. it was concluded that elevated NT-proBNP was not consistently associated with decreased LVEF [20].

All in all, there is consensus that cardiac biomarker measurements plays a role in screening for cardiotoxicity, but there is still discussion on the timing and cut-off values of cardiac biomarker measurements used as screening during anthracycline treatment.

When echocardiography is used, incorporating GLS in cardiotoxicity monitoring in conjunction with LVEF is very important as GLS may detect subclinical LV dysfunction. Sawaya et al. has shown that abnormal GLS was predictive of development of anthracycline-based cardiotoxicity 3–6 months later [21]. Although GLS was normal at the baseline echocardiogram, mid-term echocardiogram may have revealed an impaired GLS that would suggest increase risk for further LV dysfunction and support the initiation of cardioprotective medication.

Unfortunately, LVAD implantation was the sole treatment option for cardiogenic shock in this patient, because cancer progression-free survival of generally > 5 years is required for heart transplant listing [13]. Data from the EURAMOS-1 study show a 3- and 5-year cancer progression free survival after osteosarcoma diagnosis of respectively 59% and 54% [1]. Because potential donor hearts in the Netherlands are scarce this criterion is justified [22]. While chronic immune suppression is required in patients whom receive a solid organ transplantation, transplant registries in the United States show that with careful patient selection survival after a heart transplantation is similar between patients with and without a prior cancer diagnosis [23, 24]. Therefore transplantation in this population seems feasible given that there is sufficiently time period between cancer diagnosis and transplantation LVAD therapy is a viable alternative for advanced heart failure, but bleeding and mechanical complications are common in LVAD therapy with a two year survival free from disabling stroke or device removal due to malfunction of around 55–59% [25]. Therefore, in case of severe cardiotoxic complications, a comprehensive collaboration between the oncology and cardiovascular treatment teams is of paramount importance to adequately consider all options based on estimated cancer-related survival, cardiovascular status and the needs of the patient.

A limitation of this case is that earlier echocardiographic assessment of cardiac function was not performed during cancer treatment. Whereas earlier guidelines recommended cardiac evaluation after finalizing treatment in low risk patients, recent updated guidelines which were published after the treatment of this patient, recommend to perform an additional echocardiogram after 250 mg/m^2^ [10, 17].

## Conclusion

This case illustrates the importance of cardiac surveillance for cardiotoxicity—in absence of useful clinical risk factors—among AYAs with osteosarcoma and the critical implications of a cancer diagnosis on the management of advanced heart failure.

## Supplementary Information

**Additional file 1.**

## Data Availability

Supplemental video 1: echocardiography at baseline and admittance.mp4.

## Abbreviations

AYA: Adolescent and young adults
cTn: Cardiac troponin
LVEF: Left ventricular ejection fraction
NT-proBNP: N-terminal pro-brain natriuretic peptide
VA-ECMO: Veno-arterial extracorporeal membrane oxygenation
